# Supplementary material for: Chemical Compositions of Scutellaria baicalensis Georgi. (Huangqin) Extracts and Their Effects on ACE2 Binding of SARS-CoV-2 Spike Protein, ACE2 Activity, and Free Radicals
Source: Int J Mol Sci. 2024 Feb 7;25(4):2045. doi: 10.3390/ijms25042045 (PMC10888547; doi:10.3390/ijms25042045)
Supplement: Supplementary file 1 [file ijms-25-02045-s001.zip › ijms-2845069-supplementary.pdf]

**Table S1.** Chemical structures and relative ion intensity of compounds identified in Huangqin (*Scutellaria baicalensis* Georgi. root).

| ID | Retention time | | Formula | Name | Structure | Relative ion intensity (× $10^8$) | | Ref. |
|---|---|---|---|---|---|---|---|---|
| | ESI+ | ESI- | | | | WE (+/-) | EE (+/-) | |
| 1. | 12.81 | 13.16 | $C_{21}H_{22}O_{12}$ | Taxifolin 7-*O*-glucoside | 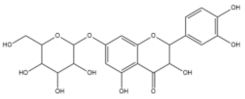 | 0.190/0.470 | nd/nd | [44] |
| 2. | 13.10 | nd | $C_{21}H_{18}O_{12}$ | Kaempferol 3-*O*-glucuronide | 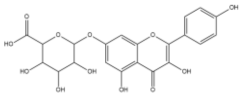 | 0.054/0.050 | nd/nd | [44] |
| 3. | 16.00 | 15.75 | $C_{21}H_{20}O_{12}$ | Carthamidin 7-*O*-glucuronide | 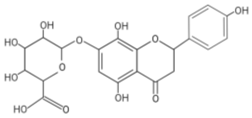 | 0.874/0.449 | 0.093/0.055 | [45] |
| 4. | 16.43 | 16.23 | $C_{15}H_{12}O_7$ | Isomer of Pentahydroxyflavanone | 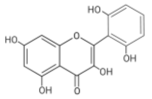 | 7.574/9.298 | 1.150/2.182 | [44] |
| 5. | nd | 18.13 | $C_{15}H_{12}O_7$ | Isomer of Pentahydroxyflavanone | 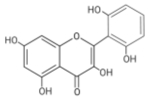 | nd/0.630 | nd/0.173 | |
| 6. | 19.59 | 19.35 | $C_{21}H_{20}O_{13}$ | 5,6,7,3′,4′-Pentahydroxy Flavanon 7-*O*-glucuronide | 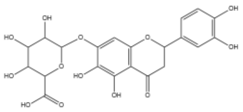 | 0.094/0.130 | nd/nd | [44] |
| 7. | 20.29 | 20.47 | $C_{22}H_{22}O_{12}$ | 5,7,2′-Trihydroxy-6-methoxyflavanone 7-*O*-glucuronide | 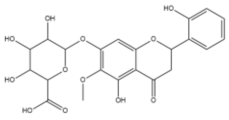 | 0.256/0.098 | 0.050/0.020 | [44] |
| 8. | 20.34 | 20.46 | $C_{15}H_{10}O_7$ | Viscidulin I | 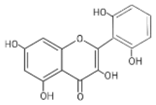 | 4.954/3.297 | 0.628/0.445 | [46] |
| 9. | 21.98 | 21.76 | $C_{21}H_{20}O_{12}$ | Isocarthamidin 7-*O*-glucuronide | 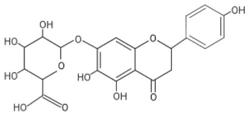 | 2.498/2.835 | 0.040/0.045 | [45] |
| 10. | 22.09 | 21.94 | $C_{16}H_{14}O_6$ | Isomer of trihydroxy-methoxyflavanone | 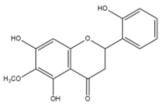 | 3.364/0.808 | 0.346/0.176 | [46] |
| 11. | 22.69 | 22.83 | $C_{21}H_{18}O_{12}$ | Scutellarin | 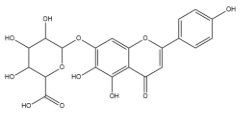 | 8.815/3.250 | 0.089/0.044 | [46] |
| 12. | 22.83 | 22.97 | $C_{21}H_{20}O_{12}$ | Eriodictyol 7-*O*-glucuronide | 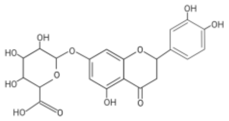 | 1.260/1.031 | 0.050/0.027 | [45] |

| | | | | | | | | |
|---|---|---|---|---|---|---|---|---|
| 13. | 22.89 | 23.06 | $C_{26}H_{28}O_{13}$ | Chrysin 6-*C*-arabinoside-8-*C*-glucoside | 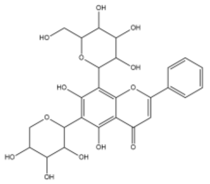 | 49.676/17.993 | 2.493/1.102 | [44] |
| 14. | 23.97 | 23.74 | $C_{26}H_{28}O_{13}$ | Chrysin 6-*C*-glucoside-8-*C*-arabinoside | 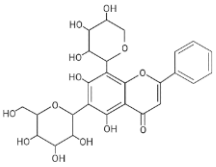 | 42.819/16.325 | 2.131/0.927 | [44] |
| 15. | 24.20 | 24.05 | $C_{15}H_{10}O_6$ | 5,7,2′,6′-Tetrahydroxyflavone | 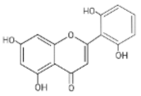 | 4.249/1.506 | 1.028/0.665 | [44] |
| 16. | 24.29 | 24.12 | $C_{15}H_{10}O_7$ | Isomer of Pentahydroxyflavone | 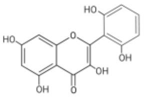 | 1.268/0.748 | 0.121/0.127 | |
| 17. | 24.56 | 24.42 | $C_{22}H_{22}O_{12}$ | 5,7,2′-Trihydroxy-8-methoxy Flavanone 7-*O*-glucuronide | 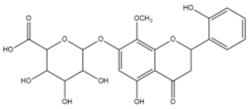 | 0.312/0.526 | 0.009/nd | [44] |
| 18. | 24.90 | 25.04 | $C_{21}H_{20}O_9$ | Isomer of Chrysin 8-*C*-glucoside | 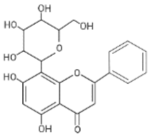 | 3.737/1.846 | 0.509/0.321 | [46] |
| 19. | 25.19 | 25.27 | $C_{15}H_{12}O_6$ | Carthamidin | 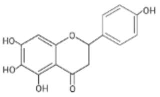 | 0.765/0.713 | 0.186/0.271 | [44] |
| 20. | 25.23 | 25.36 | $C_{22}H_{22}O_{12}$ | Isomer of trihydroxy-methoxyflavanone *O*-glucuronide | 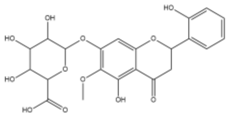 | 0.947/0.960 | 0.018/0.017 | [44] |
| 21. | 25.47 | 25.49 | $C_{16}H_{14}O_6$ | Isomer of trihydroxy-methoxyflavanone | 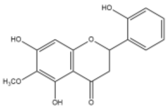 | 2.676/1.745 | 0.465/0.412 | [46] |
| 22. | 25.95 | 25.76 | $C_{22}H_{20}O_{12}$ | 5,6,7-Trihydroxy-8-methoxy-7-*O*-glucuronide | 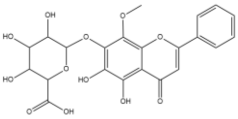 | 16.485/2.228 | nd/nd | [44] |
| 23. | 26.02 | 25.87 | $C_{15}H_{12}O_6$ | Isocarthamidin | 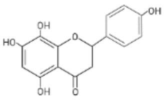 | 2.628/2.667 | 0.931/0.999 | [44] |
| 24. | 26.10 | 25.92 | $C_{22}H_{22}O_{11}$ | 5,7,2′-Trihydroxy-6-methoxyflavone 7-*O*-glucoside | 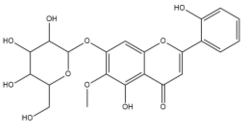 | 0.723/0.221 | 0.038/0.016 | [44] |
| 25. | 26.29 | 26.11 | $C_{17}H_{14}O_8$ | Viscidulin III | 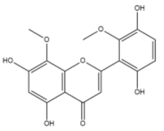 | 11.814/5.643 | 3.737/1.656 | [46] |
| 26. | 26.48 | 26.37 | $C_{15}H_{10}O_6$ | Scutellarein | 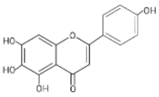 | 17.209/9.153 | 1.683/1.253 | [44] |

| | | | | | | | | |
|---|---|---|---|---|---|---|---|---|
| 27. | 26.88 | 26.97 | $C_{21}H_{18}O_{11}$ | Apigenin 7-*O*-glucuronide | 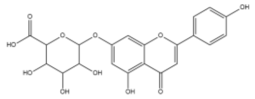 | 197.928/16.837 | 8.856/2.021 | [47] |
| 28. | 27.10 | 27.17 | $C_{21}H_{20}O_{10}$ | Apigenin 7-*O*-glucoside | 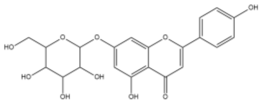 | 12.855/1.895 | 0.743/0.318 | [44] |
| 29. | 27.32 | 27.31 | $C_{21}H_{20}O_{9}$ | Isomer of Chrysin 8-*C*-glucoside | 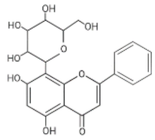 | 3.080/0.749 | 0.350/0.170 | [46] |
| 30. | 27.59 | 27.41 | $C_{22}H_{22}O_{11}$ | Isomer of trihydroxy methoxyflavone *O*-glucoside | 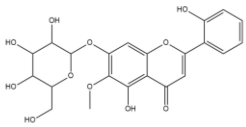 | 2.198/0.285 | 0.139/0.027 | |
| 31. | 27.72 | 27.57 | $C_{21}H_{20}O_{11}$ | Dihydrobaicalin | 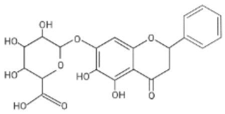 | 19.663/8.404 | 0.335/0.321 | [44] |
| 32. | 27.93 | 27.75 | $C_{22}H_{20}O_{12}$ | 5,7,8-Trihydroxy-6-methoxy Flavone-7-*O*-glucuronide | 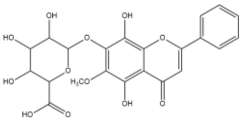 | 3.704/1.051 | nd/nd | [44] |
| 33. | 28.14 | 27.98 | $C_{22}H_{22}O_{12}$ | Isomer of trihydroxy-methoxyflavanone *O*-glucuronide | 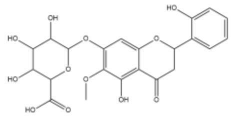 | 0.768/0.707 | 0.017/nd | |
| 34. | 28.22 | 28.09 | $C_{21}H_{18}O_{11}$ | Baicalin | 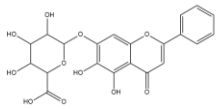 | 42.845/13.435 | 0.879/0.454 | [47] |
| 35. | 28.51 | 28.57 | $C_{21}H_{20}O_{9}$ | Chrysin 6-*C*-glucoside | 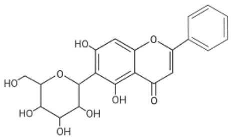 | 0.815/0.385 | 0.015/0.018 | [46] |
| 36. | 28.62 | 28.68 | $C_{16}H_{12}O_{7}$ | Pedalitin | 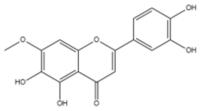 | 5.078/2.554 | 0.272/0.254 | [44] |
| 37. | 28.72 | 28.77 | $C_{21}H_{18}O_{11}$ | Norwogonin 7-*O*-glucuronide | 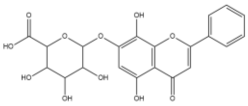 | 13.347/4.952 | 0.404/0.215 | [47] |
| 38. | 28.76 | 28.84 | $C_{22}H_{20}O_{12}$ | 5,7,2′-Trihydroxy-6-methoxy Flavone 7-*O*-glucuronoide | 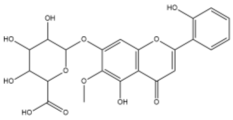 | 25.725/6.427 | 0.226/0.114 | [44] |
| 39. | 28.78 | 28.89 | $C_{16}H_{12}O_{6}$ | 4′-Hydroxywogonin | 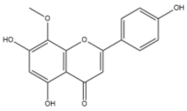 | 12.063/1.048 | 0.359/0.148 | [48] |

| | | | | | | | | |
|---|---|---|---|---|---|---|---|---|
| 40. | 28.92 | 28.97 | $C_{22}H_{22}O_{11}$ | (2S)-5,7-Dihydroxy-6-methoxyflavanone 7-*O*-glucuronide | 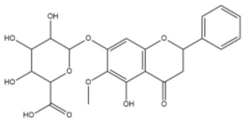 | 1.140/1.326 | 0.099/0.056 | [46] |
| 41. | 28.95 | 28.99 | $C_{21}H_{20}O_{10}$ | Baicalein 7-*O*-glucoside | 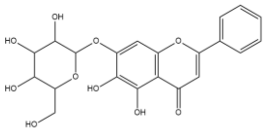 | 2.920/0.787 | 0.143/0.071 | [46] |
| 42. | 29.21 | 29.13 | $C_{21}H_{18}O_{10}$ | Chrysin 7-*O*-glucuronide | 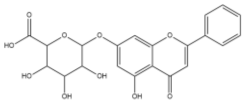 | 25.746/6.500 | 0.687/0.325 | [44] |
| 43. | 29.25 | nd | $C_{18}H_{16}O_{8}$ | Isomer of trihydroxy-trimethoxyflavone | 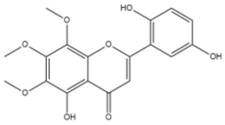 | 0.343/nd | 0.030/0.011 | |
| 44. | 29.40 | 29.23 | $C_{22}H_{20}O_{11}$ | Oroxylin A-7-*O*-glucuronide | 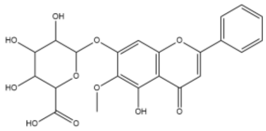 | 95.219/12.270 | 3.750/0.304 | [44] |
| 45. | 29.62 | 29.48 | $C_{22}H_{20}O_{12}$ | Isomer of Trihydroxy methoxy Flavone *O*-glucuronoide | 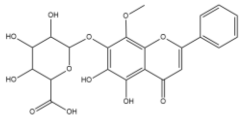 | 35.090/9.280 | 0.238/0.115 | [44] |
| 46. | 29.76 | 29.61 | $C_{15}H_{10}O_{6}$ | Isoscutellarein | 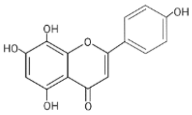 | 3.558/0.967 | 0.548/0.477 | [44] |
| 47. | 29.81 | 29.69 | $C_{21}H_{18}O_{11}$ | Baicalein 6-*O*-glucuronide | 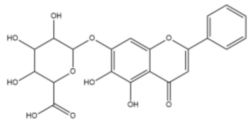 | 19.076/6.575 | 0.057/0.032 | [44] |
| 48. | 29.91 | 29.81 | $C_{17}H_{14}O_{7}$ | Isomer of trihydroxy dimethoxyflavone | 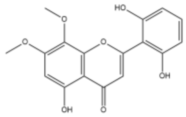 | 1.538/0.737 | 0.432/0.288 | [46] |
| 49. | 30.05 | nd | $C_{21}H_{20}O_{10}$ | Isomer of dihydroxyflavanon e *O*-glucoside | 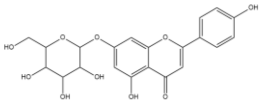 | 3.871/nd | 0.147/nd | |
| 50. | 30.08 | 30.08 | $C_{22}H_{20}O_{11}$ | Wogonoside | 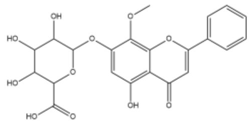 | 85.548/23.270 | 5.004/0.424 | [44] |
| 51. | 30.40 | 30.44 | $C_{17}H_{14}O_{8}$ | Isomer of Tetrahydroxy-dimethoxyflavone | 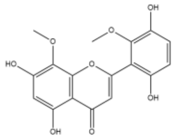 | 1.213/0.621 | 0.158/0.148 | |
| 52. | 30.72 | 30.68 | $C_{23}H_{22}O_{12}$ | 5,7-Dihydroxy-8,2′-dimethoxyflavone 7-*O*-glucuronide | 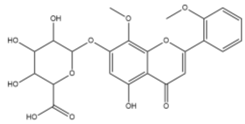 | 20.360/6.678 | 0.339/0.130 | [46] |

| | | | | | | | | |
|---|---|---|---|---|---|---|---|---|
| 53. | 31.11 | 30.98 | $C_{16}H_{12}O_6$ | Hispidulin | 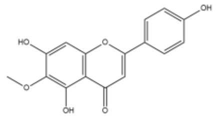 | 6.154/3.165 | 0.840/0.605 | [48] |
| 54. | 31.32 | 31.20 | $C_{18}H_{16}O_8$ | 5,2',5'-Trihydroxy-6,7,8-trimethoxyflavone | 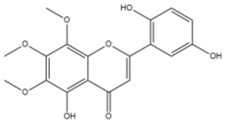 | 1.658/0.823 | 0.393/0.238 | [46] |
| 55. | 31.57 | 31.53 | $C_{17}H_{14}O_7$ | Isomer of trihydroxy dimethoxyflavone | 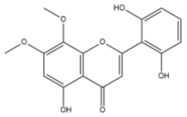 | 1.022/0.469 | 0.089/0.055 | [46] |
| 56. | 31.61 | 31.60 | $C_{16}H_{12}O_6$ | Isomer of trihydroxy-methoxyflavone | 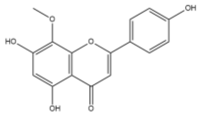 | 8.049/4.741 | 0.993/0.749 | |
| 57. | 31.84 | 31.85 | $C_{15}H_{10}O_5$ | Apigenin | 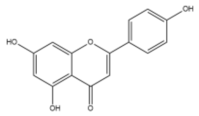 | 12.389/6.284 | 1.943/1.852 | [44] |
| 58. | 31.93 | 31.96 | $C_{17}H_{14}O_7$ | Viscidulin II | 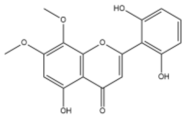 | 0.587/0.224 | 0.085/0.031 | [46] |
| 59. | 32.45 | 32.35 | $C_{17}H_{14}O_7$ | 5,7,6'-Trihydroxy-8,2'-dimethoxyflavone | 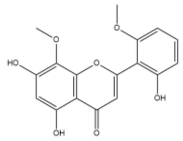 | 4.462/2.022 | 1.410/0.648 | [46] |
| 60. | 32.66 | 32.55 | $C_{18}H_{16}O_8$ | Isomer of trihydroxy-trimethoxyflavone | 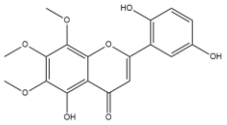 | 2.084/0.675 | 0.169/0.034 | [46] |
| 61. | 32.70 | 32.59 | $C_{16}H_{12}O_6$ | Tenaxin II | 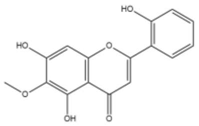 | 18.049/6.862 | 1.716/0.594 | [46] |
| 62. | 32.83 | 32.73 | $C_{15}H_{10}O_5$ | Baicalein | 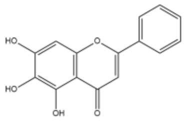 | 151.087/44.968 | 35.019/9.471 | [44] |
| 63. | 32.83 | nd | $C_{15}H_{10}O_6$ | Isomer of Tetrahydroxyflavone | 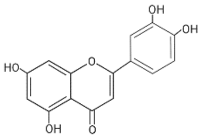 | 2.057/nd | 1.404/nd | [44] |
| 64. | 33.21 | 33.22 | $C_{17}H_{14}O_7$ | 5,8,2′-Trihydroxy-6,7-dimethoxyflavone | 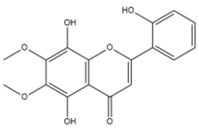 | 3.612/1.756 | 0.414/0.219 | [46] |
| 65. | 33.44 | 33.46 | $C_{16}H_{12}O_6$ | 5,6,7-Trihydroxy-4′-methoxyflavone | 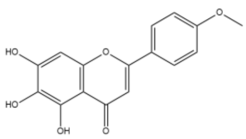 | 5.534/2.928 | 1.398/0.791 | [44, 46] |

| | | | | | | | | |
|---|---|---|---|---|---|---|---|---|
| 66. | 33.82 | 33.76 | $C_{17}H_{14}O_{7}$ | 5,7,2′-Trihydroxy-8,6'-dimethoxyflavone | 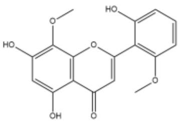 | 1.027/0.512 | 0.205/0.089 | [44] |
| 67. | 35.32 | 35.23 | $C_{17}H_{14}O_{7}$ | Isomer of trihydroxy dimethoxyflavone | 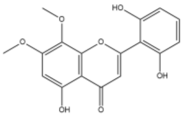 | 0.787/0.645 | 0.182/0.163 | [46] |
| 68. | 36.43 | 36.41 | $C_{18}H_{16}O_{8}$ | Isomer of trihydroxy-trimethoxyflavone | 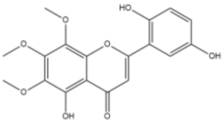 | 0.680/0.566 | 0.146/0.108 | |
| 69. | 36.90 | 36.81 | $C_{18}H_{16}O_{7}$ | Skullcapflavone | 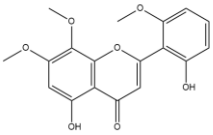 | 5.657/1.753 | 2.193/0.285 | [44] |
| 70. | 37.09 | 37.03 | $C_{16}H_{12}O_{5}$ | Wogonin | 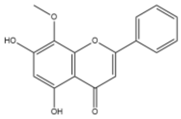 | 107.452/26.964 | 26.908/1.698 | [44] |
| 71. | 37.18 | 37.12 | $C_{15}H_{10}O_{4}$ | Chrysin | 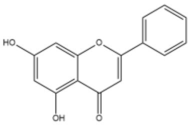 | 9.151/6.996 | 2.767/1.531 | [44] |
| 72. | 37.78 | 37.73 | $C_{17}H_{14}O_{6}$ | 5,8-Dihydroxy-6,7-dimethoxyflavone | 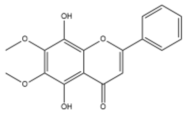 | 19.321/2.346 | 1.785/nd | [44] |
| 73. | 38.09 | 38.02 | $C_{19}H_{18}O_{8}$ | Skullcapflavone II | 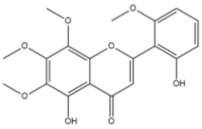 | 56.742/13.360 | 25.500/2.871 | [44] |
| 74. | 38.14 | 38.10 | $C_{16}H_{12}O_{5}$ | Oroxylin A | 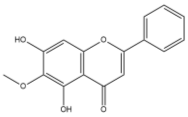 | 47.933/6.529 | 13.894/0.181 | [44] |
| 75. | 38.26 | 38.22 | $C_{17}H_{14}O_{6}$ | 5,7-Dihydroxy-6,8-dimethoxyflavone | 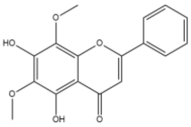 | 6.398/2.909 | 2.000/0.615 | [44] |
| 76. | 39.26 | 39.21 | $C_{18}H_{16}O_{7}$ | Tenaxin I | 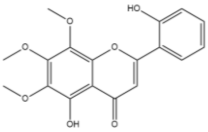 | 5.772/2.654 | 1.649/0.578 | [44] |

ESI$^+$ represents in positive mode; ESI$^-$ represents in negative mode; WE: water extracts of Huangqin; EE: ethanol extracts of Huangqin; n/d represents not detectable.
